# Improving the Specific Activity and Thermostability of Psychrophilic Xylosidase AX543 by Comparative Mutagenesis

**DOI:** 10.3390/foods11162463

**Published:** 2022-08-16

**Authors:** Kungang Pan, Zhongqi Liu, Zhengjie Zhang, Shanzheng Jin, Zhao Yu, Tianhui Liu, Tongcun Zhang, Junqi Zhao, Zhongyuan Li

**Affiliations:** 1 State Key Laboratory of Food Nutrition and Safety, College of Biotechnology, Tianjin University of Science and Technology, Tianjin 300457, China; 2 School of Chemical and Biological Engineering, Qilu Institute of Technology, Jinan 250200, China

**Keywords:** xylosidase, cold adaptation, thermostability, mutagenesis analysis

## Abstract

Improving the specific activity and thermostability of psychrophilic xylosidase is important for improving its enzymatic performance and promoting its industrial application. Herein, a psychrophilic xylosidase AX543 exhibited activity in the temperature range between 0 and 35 °C, with optimum activity at 20 °C, which is lower than that of other reported psychrophilic xylosidases. The thermostability, specific activity, and catalytic efficiency of the site-directed variants G110S, Q201R, and L2 were significantly enhanced, without affecting the optimal reaction temperature. Comparative protein structural analysis and molecular dynamics simulation indicated that these improvements might be the result of the increased hydrogen bonds interaction and improved structural rigidity. Furthermore, homologous module substitution with four segments demonstrated that the psychrophilic characteristics of AX543 are the results of the whole protein structure, and the C-terminal segment A4 appears to be more essential in determining psychrophilic characteristics, exhibiting potentiality to produce more psychrophilic xylosidases. This study provides valuable structural information on psychrophilic xylosidases and also offers attractive modification strategies to modify catalytic activity, thermostability, and optimal reaction temperature.

## 1. Introduction

β-xylosidase (EC 3.2.1.37) is a rate-limiting enzyme in the xylan enzyme degradation system, which can completely decompose xylan by synergistic action with xylanase [1]. Xylosidase has been widely used in food processing, such as for reducing the viscosity and turbidity of beer and wine, promoting the liquefaction of coffee mucus, improving the extraction rate of spices and dyes, and producing xylose and other high value-added products such as xylitol [2]. In order to reduce the loss of active ingredients, flavor substances, and nutrients in food products, some food processing procedures, such as wine fermentation and fruit juice clarification, usually need to be carried out at low temperatures (<30 °C). However, the vast majority of discovered xylosidases belong to medium-high temperature enzymes, with the optimal reaction temperature from 40 °C to 60 °C, and their activities at low temperature conditions are very weak, or even completely lost (http://www.brenda-enzymes.org (accessed on 15 June 2022)) [3]. Psychrophilic xylosidases might provide a promising new way to solve this problem, since they have high catalytic activity at a low temperature range, with the optimal reaction temperatures below 30 °C [4]. So far, only a few psychrophilic xylosidases have been previously reported, as shown in Table 1 [5,6,7,8,9,10,11]. Our group has been exploring psychrophilic xylosidases from different environments, and reported a novel psychrophilic xylosidase AX543 from *Acremonium* sp. WCQ6A after the activity screening of many xylosidase candidates [12].

In order to display efficient catalytic activity in cold conditions, protein structures of psychrophilic enzymes must be flexible. Previous studies have shown that the flexible protein structure of psychrophilic enzymes was likely damaged during enzyme processing; thus, the commercial application of these reported psychrophilic xylosidases were limited by their poor thermostability and low specific activity [4]. To overcome this disadvantage, one strategies is to continuously isolate more psychrophilic xylosidases from the natural environment and gene databases, but this is still labor and time consuming, as well as full of uncertainty [13]. Instead of enzyme mining, desired enzymes can be generated by enzyme engineering based on the relationship between protein structure and function [14]. It has generally been assumed that mesophilic and thermophilic enzymes have a tendency to be more rigid, whereas psychrophilic enzymes are associated with structure flexibility [4]. Compared with mesophilic and thermophilic enzymes, psychrophilic enzymes subtly adjust their amino acid composition, e.g., by increasing the content of glycine, reducing the content of proline and arginine, and developing loop regions [13,14,15,16,17]. For example, the ratios of proline and arginine in cold-active valine dehydrogenase from *Cytophaga* sp. KUC-1 (2.16% and 2.97%) are lower than those of medium temperature valine dehydrogenase from *Streptomyces coelicolor* (4.13% and 6.06%) and *Streptomyces fradiae* (4.32% and 7.84%) [18]. The cold-active amylase AHA has less arginine (2.9% vs. 5.6%) and prolines (2.9% vs. 4.2%) compared with the thermophilic amylase PPA [19]. Besides, psychrophilic dehydrogenase also possesses lower amounts of arginine and higher amounts of glycine in comparison with its mesophilic and thermophilic homologs [20].

Among reported psychrophilic xylosidases, AX543 exhibited the lowest optimal reaction temperature (20 °C, Table 1). Notably, a thermophilic xylosidase Xyl43A (AHC72382.1) from *Humicola insolens*, sharing the highest amino acid sequence identity (80%) with AX543, displayed its optimum reaction temperature at 50 °C [21]. Herein, based on the multi-sequence alignment, molecular dynamics simulation, and rational/semi-rational mutagenesis analysis of these two xylosidases, this comparative study has enabled us to identify possible key factors affecting the cold adaptation of xylosidase AX543, including the related regions, amino acid sites, and molecular bond forces. Moreover, the specific activity and thermostability of AX543 were further improved by reducing the structural flexibility.

## 2. Materials and Methods

### 2.1. Strains, Plasmids, and Biochemical Reagents

The plasmids were amplified with *Escherichia coli* DH5α and expressed with *E. coli* BL21 (DE3) as the prokaryotic host. *E. coli* strains were cultured in LB medium (0.5% *w*/*v* yeast extract, 1% *w*/*v* peptone, and 1% *w*/*v* NaCl), with kanamycin (100 µg/mL) added to the LB medium. Phusion DNA polymerase, restriction endonuclease, and T4 DNA ligase were provided by Thermo Fisher Scientific (Shanghai, China). DNA purification kits and plasmid extraction kits were purchased from Tiangen (Beijing, China). Substate 4-nitrophenyl-β-D-xylopyranoside (pNPX) was purchased from Sigma (St. Louis, MO, USA). Other chemical reagents were of analytical grade and commercially available.

### 2.2. Protein Sequence and Structure Analysis

The amino acid sequence of AX543 was compared with other GH43 family xylosidases using clustalW software (https://www.genome.jp/tools-bin/clustalw (accessed on 10 March 2022)). The amino acid composition of psychrophilic, mesophilic, and thermophilic counterparts was calculated using Vector NTI software (version 10.0, Invitrogen, Carlsbad, CA, USA). The protein structures of psychrophilic xylosidase AX543, thermophilic xylosidase Xyl43A, and the variants were predicted using SWISS-MODEL (https://swissmodel.expasy.org/ (accessed on 20 May 2022)), and then the protein structures and intermolecular forces were analyzed by PyMOL software (https://pymol.org/2/ (accessed on 25 May 2022)). The root mean square deviation (RMSD) between two proteins was calculated by PyMOL software (accessed on 22 July 2022).

### 2.3. Molecular Dynamic Simulation

Molecular dynamic (MD) simulation was performed to compare the dynamic properties of AX543 at 300 K and 350 K using GROMACS 4.5.5 package with the AMBER99SB force field. The initial model of AX543 was solvated with a box of TIP3P water molecules; then the energy minimizations were carried out with 2000 steps of steepest descent followed by 2000 steps of conjugate gradient. For the next MD simulation steps, the system was gradually heated and equilibrated, and the simulation time was 10 ns. Each simulation was repeated three times, with the same initial configurations. The root means square fluctuation (RMSF) was calculated for the protein backbone atoms using least-squares fitting.

### 2.4. Site-Directed Mutagenesis

The gene sequences of wild-type AX543 and Xyl43A, respectively, were amplified with primers (Table S1). The PCR fragments were digested by *EcoR* I and *Not* I, ligated into pET-28a(+) vector, and transformed into *E. coli* BL21(DE3). In order to obtain the site-directed variants, a series of PCR products of the variants were amplified by whole plasmid PCR with the corresponding primers and the plasmid of pET-AX543 as a DNA template (Table S1). After removing the template plasmid DNA digested by *Dpn*I, the PCR products were transformed into *E. coli* BL21(DE3) to produce recombinant variant plasmids pET28-G110S, pET28-G216A, pET28-A119P, pET28-K125P, pET28-K285R, pET28-Q201R, pET28-L1, pET28-L2, pET28-L3, and pET28-L4.

In order to evaluate the effects of four segments (A1, A2, A3, and A4) on the optimal reaction temperature of AX543, the 10 chimeric variants (R1–R10) were constructed by overlap PCR using wild-type AX543 or Xyl43A as a template. The final PCR fragments of the correct size were digested by *EcoR* I and *Not* I and ligated into the pET-28a(+) vector, and were transformed into *E. coli* BL21(DE3). All constructed variants were confirmed by DNA sequencing (GENEWIZ, Suzhou, China).

### 2.5. Expression and Purification of Wild-Type and Variants

The wild-type and the variants were incubated in LB medium containing 100 μg/mL kanamycin at 37 °C for 2 h, and then were induced by the addition of 1 mM Isopropyl-β-D-thiogalactopyranoside (IPTG) for 12 h at 25 °C. In order to obtain the recombinant protein, the variants were harvested by centrifugation at 12,000× *g* (4 °C for 15 min) and resuspended in 10 mM phosphate buffer (8 g/L NaCl, 3.58 g/L Na_2_HPO_4_·12H2O, 0.2 g/L KCl, 0.27 g/L KH_2_PO_4_, pH 7.4). After being disintegrated by an ultrasonic cell disintegrator (Ningbo Xinzhi Instrument, Ningbo, China), the intracellular crude proteins were obtained by further centrifugation at 12,000× *g* (4 °C for 10 min) to remove the cell debris. The recombinant proteins were purified by nickel affinity chromatography (GE Health-care, Uppsala, Sweden) with 10 to 500 mM imidazole in buffer (20 mM Tris-HCl, 50 mM NaCl, pH 8.0). The protein fractions corresponding to different imidazole concentrations were collected and subjected to sodium dodecyl sulfate (SDS)-polyacrylamide gel electrophoresis. The protein concentration was determined using a protein assay kit (Bio-Rad, Hercules, CA, USA).

### 2.6. Enzyme Activity Assays and Biochemical Characterization

To determine the xylosidase activity of the wild-type and variants, each reaction system contained 450 μL of *p*NPX substrate (2 mM) and 50 μL of protein sample (2 μM); the releasing para-nitrophenol (*p*NP) was determined to occur at 405 nm by spectrophotometer (MD SpectraMax190, Chatsworth, CA, USA). The optimum temperature for AX543 activity was determined at different temperatures, between 0–50 °C, and a pH of 6.0. The optimal pH for enzyme activity was measured using 0.2 M citric acid-Na_2_HPO_4_ buffer (pH 4.0–8.0) or 0.2 M glycine-NaOH buffer (pH 8.0–9.0) at the optimum temperature. After incubation at 40 °C for different periods of time, the thermal stabilities of the enzymes were determined by measuring the residual activity at pH 6.0 and 20 °C.

The specific activities of wide-type AX543 and its variants were assayed under the optimum reaction conditions. The kinetic parameters of wide-type AX543 and its variants were determined in McIlvaine buffer containing 450 μL *p*NPX (0.1 to 2 mM) with 50 μL of 2 μM protein sample at 20 °C and pH 6.0 for 5 min. *K*_m_ and *V*_max_ values were determined on the basis of Lineweaver–Burk plots using GraphPad Prism software (Version 5.0, GraphPad Soft-ware Inc., La Jolla, CA, USA), and all the experiments were carried out three times.

## 3. Results and Discussions

### 3.1. Expression and Biochemical Characterization of Xylosidase AX543 in E. coli

**Figure 1:**
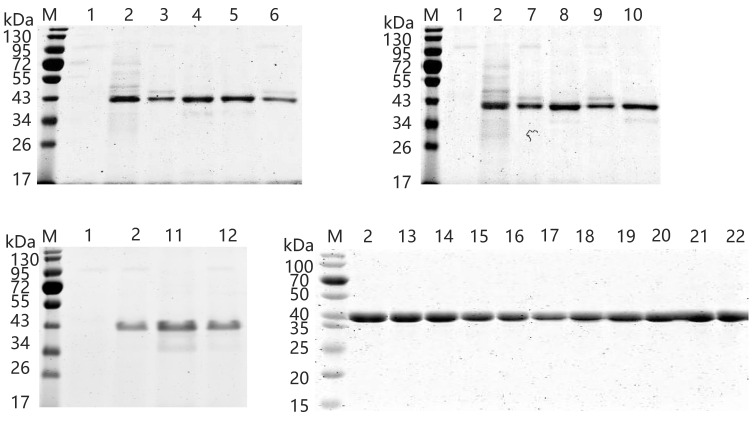
SDS-PAGE analysis of purified recombinant proteins of wild type AX543 and its variants. Lane M, molecular mass marker; Lane 1, the intracellular supernatant of plasmid pET28a(+) expressed in *E. coli*; Lane 2, wild type AX543; Lane 3, mutant L1; Lane 4, mutant L2; Lane 5, mutant L3; Lane 6, mutant L4; Lane 7, mutant K125P; Lane 8, mutant A119P; Lane 9, mutant G216A; Lane 10, mutant G110S; Lane 11, mutant Q201R; Lane 12, mutant K285R; Lane 13, chimera R1; Lane 14, chimera R2; Lane 15,chimera R3; Lane 16, chimera R4; Lane 17, chimera R5; Lane 18, chimera R6; Lane 19, chimera R7; Lane 20, chimera R8; Lane 21, chimera R9; Lane 22, chimera R10.

**Figure 2:**
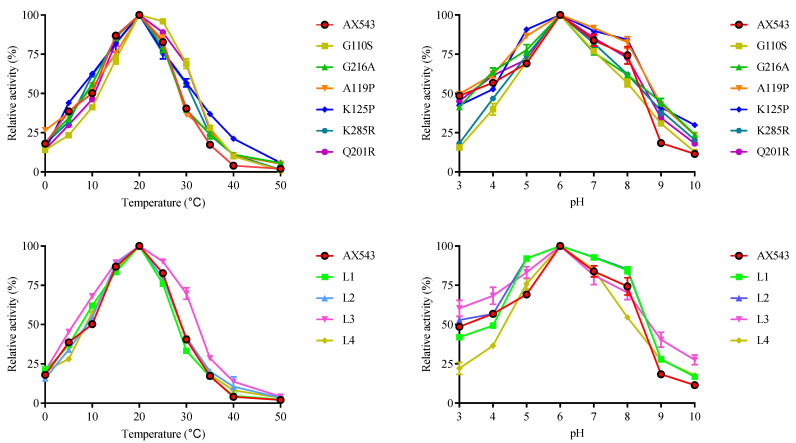
The effects of temperature and pH on the wild-type AX543 and its variants.

**Figure 3:**
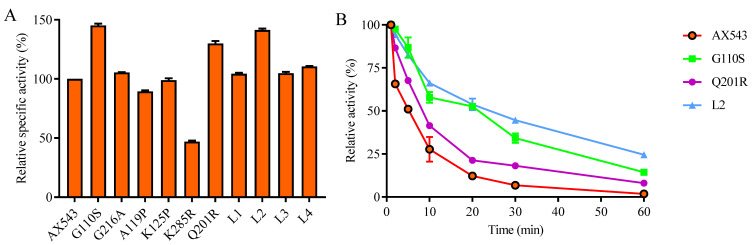
The specific activities (**A**) and thermostability (**B**) of the wild-type AX543 and its variants.

Psychrophilic xylosidases have great potential for food and other industries requiring low temperature conditions, and a psychrophilic xylosidase AX543 was previously cloned and expressed in *Komagataella phaffii* by our research group [12]. In this study, in order to facilitate the following mutagenesis of AX543, the gene of AX543 was successfully expressed in *E. coli* BL21(DE3) because of its fast growth rate and easy molecular operation. After inducing the recombinant protein with IPTG, the hydrolysis activity of crude enzyme AX543 was detected, and no activity was shown in the cultures harboring an empty plasmid pET-28a(+). The recombinant protein of AX543 was purified to homogeneity at 200 mM imidazole by Ni-affinity chromatography. The SDS-PAGE gel showed a single band of molecular mass of approximately 42 kDa (Figure 1), which is consistent with its calculated molecular mass. The optimal pH of AX543 is 6.0 (Figure 2). AX543 exhibited its hydrolytic activity from 0–35 °C, with its maximum activities at 20 °C, and still retained 38.64% and 18.02% relative activity at 5 °C and 0 °C, respectively (Figure 2). Among the reported psychrophilic xylosidases, AX543 has the lowest optimal reaction temperature (Table 1). Besides, similar to the other psychrophilic enzymes [3,4], AX543 displayed poor thermostability (Figure 3), only retaining about 51.05% of its initial activity at 40 °C for 5 min, and losing all activity at 40 °C for 1 h. Notably, 8 out of 10 reported psychrophilic xylosidases all belong to GH43 family, which suggests that the GH43 family might be a desirable enzyme database for mining cold-active xylosidases (Table 1).

### 3.2. Improvement of Specific Activity and Thermostability by Site-Directed Mutagenesis

Previous studies reported that psychrophilic enzymes subtly adjust their amino acid composition and reduce intramolecular interactions (such as hydrogen bonds, salt bridges, and disulfide bonds), so that their protein structure and active sites become more flexible and can better adapt to a low temperature environment [3]. Based on the multiple sequence alignment with other reported xylosidases from databases and the literature, thermophilic xylosidase Xyl43A (from *Humicola insolens*) and PtXyl43 (from *Paecilomyces thermophila*) were found to share a high amino acid sequence identity of 80% and 67%, respectively, with psychrophilic xylosidase AX543 [22]. The three-dimensional structures of low-temperature xylosidase AX543, high-temperature xylosidases Xyl43A, and PtXyl43 (with 80% shared identity) were established using xylosidase RS-223 (PDB:4MLG) as a template. The similarities of these xylosidases AX543, Xyl43A, and PtXyl43 to the template RS-223 are 65%, 68%, 64%, and the identities are 56%, 59%, 53%, respectively. Besides, GMQE and QMEANDisCo global scores are parameters which provide an overall model quality measurement between 0 and 1, with higher numbers indicating higher expected quality. The GMQE and QMEANDisCo global scores for the three-dimensional structural models are all above 0.8, which suggests that they are of high quality. The enzymatic property of xylosidase RS-223 has not yet been reported.

**Figure 4:**
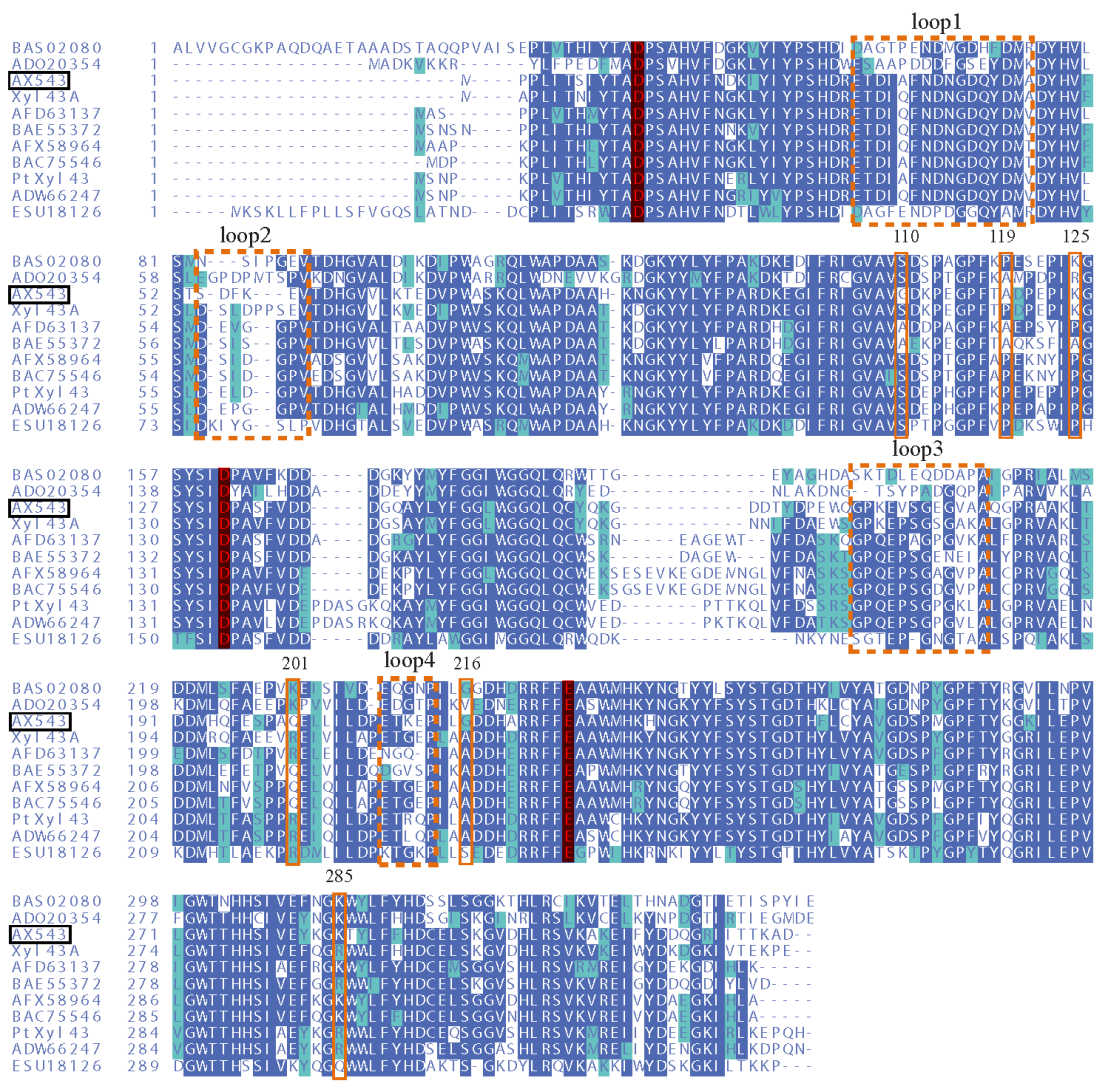
The deduced amino acid sequence alignment of AX543 (ON730957) and the reference family GH43 β-xylosidases. They are Xyl43A from *Humicola insolens* (AHC72382.1), PtXyl43 from *Paecilomyces* (ADM33794.1), and β-xylosidases from *Aspergillus terreus* (AFD63137.1), *Thermomyces lanuginosus* (ADW66247.1), *Talaromyces purpureogenus* (AFX58964.1), *Aspergillus oryzae* RIB40 (BAE55372.1), *bacterium* (BAS02080.1), *rumen bacterium* (ADO20354.1), *Penicillium herquei* (BAC75546.1), and *Fusarium graminearum* PH-1 (ESU18126.1). The identical amino acids are marked with dark and light blue backgrounds. Three conserved catalytic residues of AX543 (Asp12, Asp131, and Glu225) are marked in red letters, and the mutation sites and mutation loop regions are labeled with a solid orange frame and a dotted orange frame, respectively.

Compared with thermophilic xylosidases Xyl43A and PtXyl43, AX543 has a higher glycine ratio than Xyl43A and PtXyl43, and AX543 contains extra glycine at the 110 and 216 sites (Figure 4). Moreover, the ratio of proline in AX543 is lower than in Xyl43A and PtXyl43 (Table 2), the corresponding site of 119 A of AX543 is proline 123P in Xyl43A, and the corresponding site of 125 K of AX543 is proline 129 P in PtXyl43 (Figure 4). Furthermore, as shown in Table 2, the number of total hydrogen bonds and arginine-mediated hydrogen bonds of AX543 were less than that of high temperature xylosidase Xyl43A. The arginine/lysine ratio of AX543 is also lower than that of Xyl43A and PtXyl43. It was determined that the corresponding site of 201 in AX543 is arginine in Xyl43A, and the corresponding site of 285 in AX543 is also arginine (Figure 4).

To explore the effects of glycine, proline, and arginine on the psychrophilic characteristics of AX543, the corresponding single site-directed variants designed as G110S, G216A, A119P, K125P, Q201R, and K285R were successfully constructed and expressed (Figure 1). All these variants exhibited their highest activity at 20 °C (Figure 2), which is identical to the wild type. Notably, AX543 lost nearly all activity after 1 h at 40 °C, and the thermostability of G110S and Q201R were significantly improved (Figure 3), retaining 14.36% and 8.02% relative activity, respectively, after 1 h at 40 °C. Moreover, the specific activities of G11 and Q201R increased to 145.17% and 130.00%, respectively, of the wild type (Figure 3). The catalytic efficiencies (*k*cat/*K*m) of 201R, G110S, and L2 increased to 9.49, 7.23, and 8.12 (1/s·mM), respectively, and the catalytic efficiency (*k*cat/*K*m) of wild type AX543 is only 4.64 (1/s·mM).

**Figure 5:**
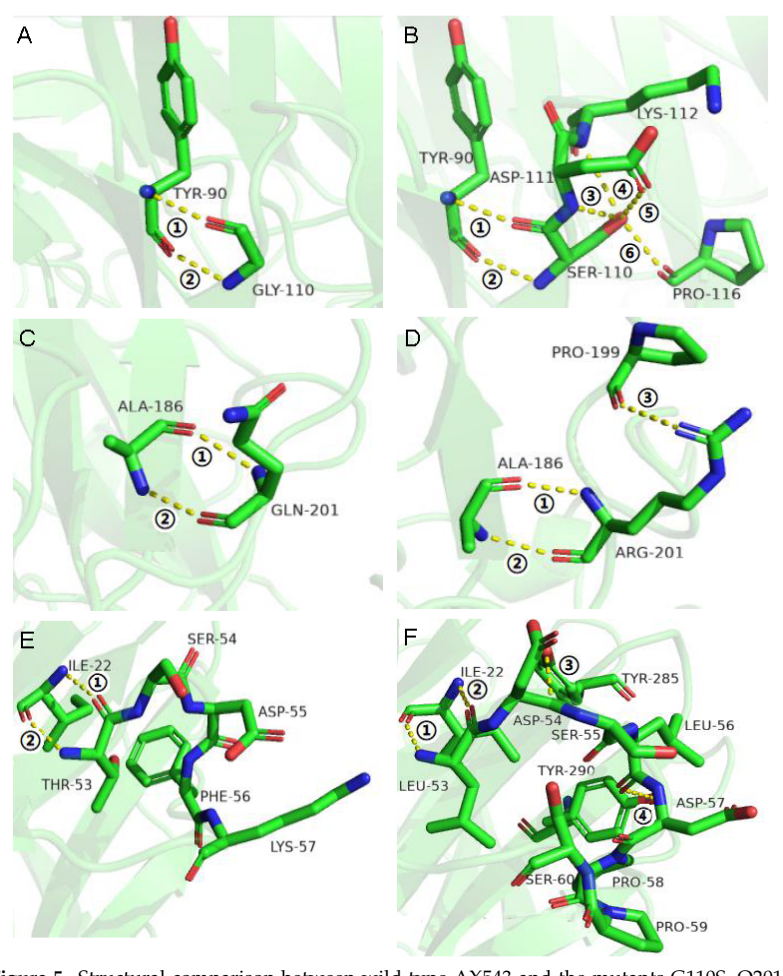
Structural comparison between wild type AX543 and the mutants G110S, Q201R, and L2. (**A**) G110 of AX543; (**B**) S110 of mutant G110S; (**C**) Q201 of AX543; (**D**) R201 of mutant Q201R; (**E**) Loop2 of AX543; (**F**) Loop2 of mutant L2. The yellow dotted line represents the hydrogen bonds. The different hydrogen bonds were marked with ①, ②, ③, ④, ⑤, ⑥.

Further intermolecular interaction analysis showed that G110S has four more hydrogen bonds with the surrounding sites than AX543 (6 vs. 2), and Q201R has one more hydrogen bond than the wild-type (3 vs. 2) (Figure 5). These introduced hydrogen bonds might reduce the flexibility of the protein structure and improve their thermostability [3]. These results showed that reduced glycine and increased arginine are applicable for improving the thermostability for xylosidases without affecting the optimal reaction activity. Previous studies have also reported that increasing the overall rigidity of protein, while minimizing its influence on the active site could improve thermostability, either without changing catalytic activity, or ideally, while positively promoting the catalytic activity of phytase, β-glucanase, and xylanase [23].

### 3.3. Effects of Flexible Loop Regions on Cold-Adaptation of AX543

Among the potential affecting factors, loop region is assumed as one of the main factors leading to the flexibility of the enzyme molecular structure, including the differences in number, length, amino acid composition, and molecular inter-atomic forces in the loop regions [24]. For example, loop L3 of low-temperature glucosidase BglU is crucial to its cold-active characteristics [25]. Low-temperature xylosidase XynGR40 has more loop regions than medium-temperature xylanase, as identified through crystal structure analysis [26,27]. The thermostability of endoglucanase decreases after the β-sheet is mutated into the loop region [28]. Moreover, loop regions are also involved in the formation of substrate binding sites and active sites, thus affecting enzyme characteristics. The loop region of GH11 xylanases involves substrate recognition and catalysis [29], and the loop regions of endoglucanases and polygalacturonases can also modulate their catalytic efficiency [30,31].

**Figure 6:**
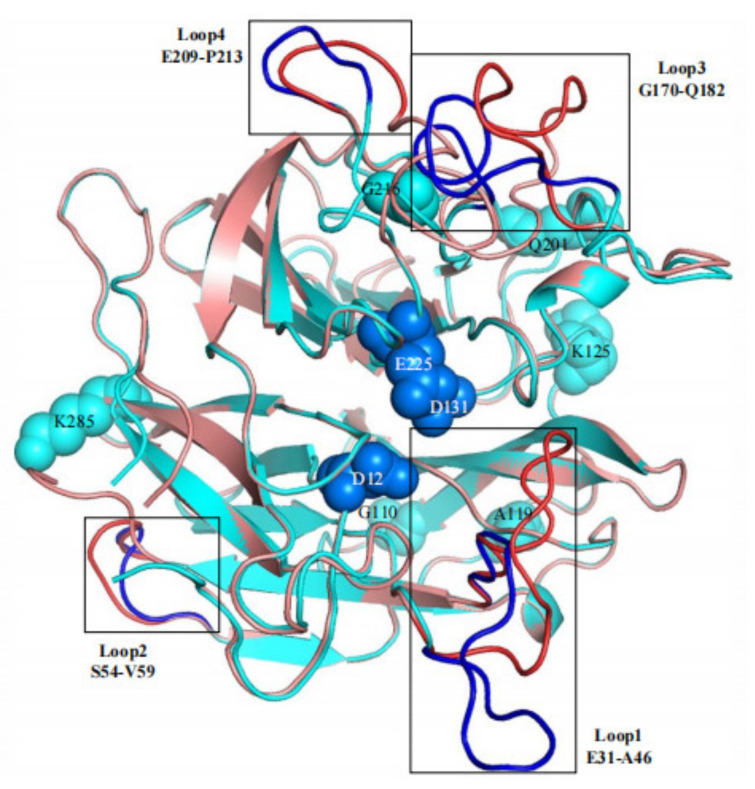
The comparison of protein structures between xylosidase AX543 (blue) and the xylosidase Xyl43A (red). Three conserved catalytic residues of AX543 (Asp12, Asp131, and Glu225) are marked in navy blue, and the single mutation site is displayed in light blue. The mutation loop regions are shown by deep color and marked with a border.

**Figure 7:**
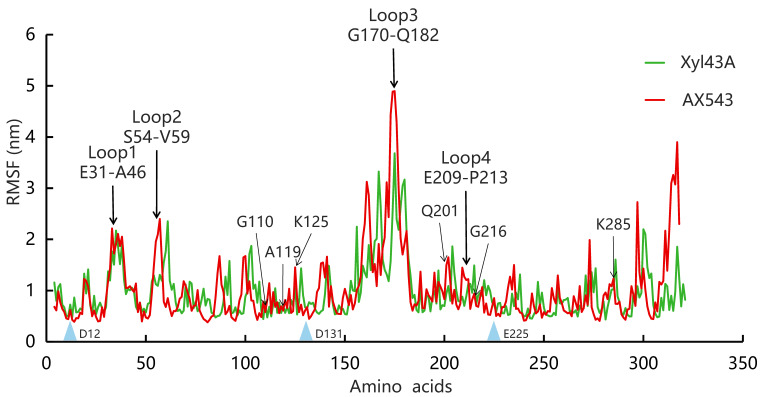
Molecular dynamics simulation and root mean square fluctuation (RMSF) of AX543 (red) and Xyl43A (green). The three conserved AX543 catalytic residues (Asp12, Asp131, and Glu225) are marked with blue triangles.

In this study, based on the superimposed structural analysis between xylosidase AX543 and its mesophilic and thermophilic counterparts Xyl43A and PtXyl43, four significantly different loop regions were identified, including loop1 (E31-A46), loop2 (S54-V59), loop3 (G170-Q183), and loop4 (E209-P213) of AX543 (Figure 6). The corresponding protein structures of these four regions in the template for xylosidase RS-223 are also loops. Molecular dynamics simulation of AX543 and high-temperature enzyme Xyl43A were further comparatively analyzed at 300 K by Gromacs software, and the flexible variation of the whole protein structures was analyzed using the average RMSF value (Figure 7). RMSF value refers to the average amplitude of each atom relative to the reference position and reflects the overall flexible state of the system; the larger value represents the stronger amino acid flexibility of the protein [32]. Compared with xylosidase Xyl43A, the molecular structures of loop1, loop2, and loop3 regions of AX543 exhibited higher RMSF values than those of the corresponding regions in Xyl43A, perhaps indicating the conformation instability of these four loop regions. Consequently, to validate the effects of the loop region, the four loop regions of AX543 were substituted with the corresponding region of Xyl43A to produce the variants of L1, L2, L3, and L4, respectively, and these four variants were further successfully constructed, expressed, and purified in *E. coli* (Figure 1).

The optimal temperatures of variants L1, L2, L3, and L4 were found to still be 20 °C, which is identical to that of the wild type. Only the hybrid enzyme L2 was more stable than the wild type, which retained 82.71% relative activity at 40 °C for 5 min and 52.25% relative activity at 40 °C for 20 min, respectively (Figure 3). Moreover, the specific activity of L2 significantly increased to 141.31% of the wild type enzymes (Figure 3), and the catalytic efficiency also increased (Table 3). Further intermolecular force analysis indicated that the number of hydrogen bonds in the loop2 region increased compared with the wild type, and the number of internal hydrogen bonds in the loop2 region was identical to the wild type, but another 2 hydrogen bonds were found between the loop2 region of Y285 and Y290 in AX543 (Figure 5). This increased interaction is beneficial to maintain the structural stability of the L2 region, thus reducing the fluctuation of main chain atoms. Previous studies reported that introducing hydrogen bonds in the enzyme protein molecules can increase the energy barrier at high temperatures, further improving the enzyme activity and thermal stability [33].

### 3.4. Homologous Module Substitution between Psychrophilic and Thermophilic Xylosidases

In this study, the rational site-directed amino acid and short loop region variants of AX543 resulted in three variants exhibiting improved thermostability, without any change in the optimal reaction temperature. Thereby, it is speculated that the low optimal reaction temperature of AX543 is not due to a single amino acid site or loop region, but a network of multiple regions. In order to obtain more structural information regarding the low optimal reaction temperature of xylosidase AX543, semi-rational homologous module substitution was applied in the following research. The homologous substitution strategy has been previously applied to various proteins, which can lead to the design of proteins with enhanced biological performance to meet the requirements of specific research and technological goals [34,35]. Previously, after fusion of the thermostabilizing domain A2 of xylanase XynA to the N-terminal region of Xyn2, the optimal temperature of the hybrid enzyme was 10 °C higher than for Xyn2, and its thermostability was also improved [36]. The multiple substitutions between two xylanases produced by *Streptomyces lividans* has shown that the C-terminal region of xylanase plays an important role in determining the optimal temperature [37].

**Figure 8:**
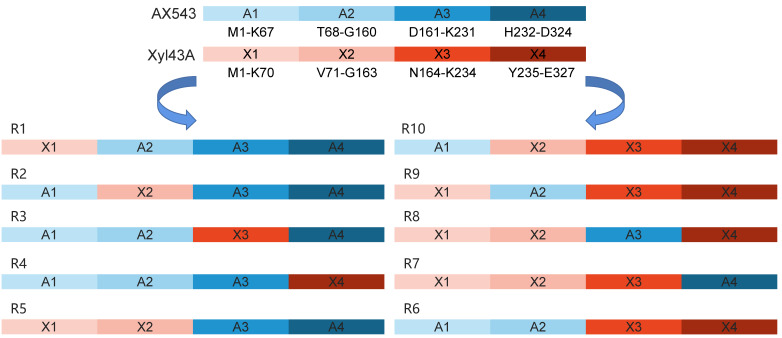
The schematic diagram of the molecular construction of chimeras.

In this study, the amino acid sequences of psychrophilic xylosidases AX543 and thermophilic xylosidases Xyl43A have significant homology (the highest identity at 80%), but quite different optimal temperatures (20 °C vs. 50 °C). Previous studies also reported that thermophilic and mesophilic isoenzymes have high homology (40–80%), but often exhibit different performances, including thermal stability and catalytic efficiency [38]. To further analyze the cold-active property, as well as to study the effect of the exchange of homologous segments, the whole sequence of AX543 was divided into four segments: A1 (M1-K67), A2(T68-G160), A3 (D161-K231), and A4 (H232-D324) (Figure 8). This design strategy was based on the length, the position of three active sites, and the secondary structures, which ensures the preservation of the catalytic domain and catalytic activity. Among these four segments, segment A1 contained the active site of D12, loop1, and loop2 region; segment A2 contained active site of D131; segment A3 contained the active site of E225, loop3, and loop4. There was no active site in segment A4 (Figure 4). As shown in Figure 8, the chimeric enzymes designated as R1-R10 were constructed by substituting segments from A1-A4 segments of the AX543 gene with corresponding X1-X4 segments of Xyl43A and in turn, substituting the Xyl43A gene with the corresponding segments of AX543. Finally, these 10 hybrid genes, R1-R10, were successfully constructed and expressed in *E. coli* (Figure 1).

**Figure 9:**
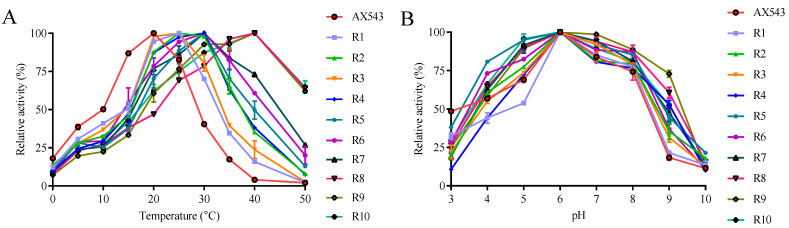
The effects of temperature (**A**) and pH (**B**) on AX543 and its chimeras.

As shown in Figure 9, all of the optimal temperatures of chimeras were admixtures of those of two parents with the range of 25–40 °C (Figure 9A), and the optimal pH for all of the chimeras is 6.0 (Figure 9B). Chimeras R1, R2, and R3 showed their optimal activity at 25 °C; R4, R5, R6, R7 showed their optimal activity at 30 °C; and R8, R9, and R10 displayed their maximum activity at 40 °C. Three novel psychrophilic xylosidase—R1, R2, and R3—were produced, which suggests the generality of the cold-active related regions of AX543, to some extent. Notably, the optimal temperatures of chimeras R8 (with A3), R9 (with A2), R10 (with A1) are 10 °C lower than that of Xyl43A (50 °C), respectively, and the optimal temperature of chimeras R7 is 20 °C lower than that of wild type Xyl43A. These results showed that the low optimal reaction temperature of AX543 is the result of a network of four segments, and the C-terminal segment A4 of AX543 seems to be more important for its low optimal reaction activity than the other three segments.

**Figure 10:**
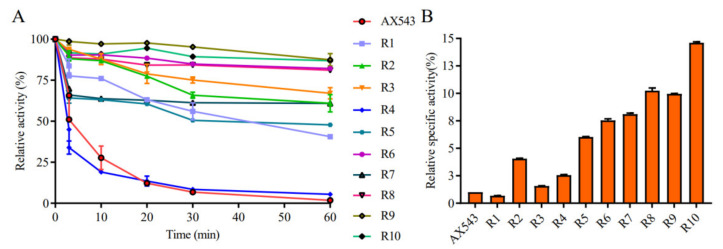
The thermostability (**A**) and specific activities (**B**) of purified xylosidase AX543 and its chimeras.

Moreover, compared to AX543, the thermostability of the chimeras, except for R4, was largely improved (Figure 10A). Chimeras R6 (with A1A1), R8 (with A3), R9 (with A2), and R10 (with A1) retained above 81% of relative residual activity after incubation at 40 °C for 60 min, which is similar to the results for Xyl4, while R7 (with A4) only showed 63.79% of relative residual activity after incubation at 40 °C for 60 min, which suggests that the C-terminal segment A4 has the highest impact on the poor thermostability of psychrophilic xylosidase AX543.

The specific activities of all variants were compared at both the optimal reaction temperature and low temperature, and it was found that the specific activities of all chimeras, except for R1, increased significantly (Figure 10B). Specifically, the particular activities of R10 at 20 °C were 14.66 fold that of AX543. In addition, the catalytic efficiencies (*K*cat/*k*m) of these variants, except for R1 and R4, were improved, as shown in Table 4. The enhanced specific activity and catalytic efficiency of the variants by segment substitution might be due to the high specific activity and catalytic efficiency of Xyl43A. This result suggests that segment substitution is an effective strategy for acquiring novel psychrophilic xylosidases.

**Figure 11:**
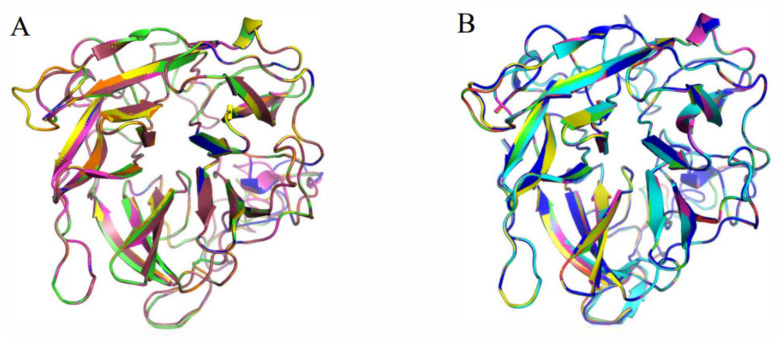
The comparison of protein structures between the variants and xylosidase AX543 and Xyl43A. (**A**) R1 (orange), R2 (green), R3 (brown), R4 (pink), R5 (yellow), and AX543 (blue); (**B**) R6 (light blue), R7 (yellow), R8 (navy blue), R9 (green), R10 (pink), and Xyl43A (red).

In addition, the 3D structure of its variants are all modeled and aligned with wide-type (AX543 and Xyl43A, Figure 11). The RMSD value can reflect the differences between two protein structures. Interestingly, the RMSD values between four variants (R6, R7, R9, or R10) and Xyl43A are higher than the RMSD values between them and AX543 (Table 5), which means their protein structures are more similar to thermophilic xylosidase Xyl43A. while, the RMSD values between R1, R2, R4, R5, and Xyl43A are lower than the RMSD values between them and AX543 (Table 5), which shows their protein structures are more similar to psychrophilic xylosidase AX543. To some extent, these results are basically consistent with their optimal reaction temperature, specific activity, and thermostability.

## 4. Conclusions

In this study, the thermal stability and activity of psychrophilic enzymes were significantly improved by mutagenesis, and C-terminal segment A4 of AX543 appears to be more essential in determining its psychrophilic characteristics. This study provides insight into the cold adaption structural characteristics of psychrophilic xylosidases and also facilitates the further molecular design of novel potential psychrophilic xylosidases.

## Figures and Tables

**Table 1:** Enzymatic properties of the reported psychrophilic xylosidases.

Microorganism or Environment	Protein Name	GenBank Accession No.	Family	Optimal pH	Optimal Temperature	Reference
*Acremonium* sp. *WCQ6A*	AX543	ON730957	GH43	6	20	This study
*Fusarium graminearum*	Xylo	ESU18096	GH43	6	20	[5]
*Bacillus*	rHJ14GH43	KY391885	GH43	7	25	[6]
*Rhizophlyctis rosea*	RrXyl43A	AYV64572	GH43	7	25	[7]
*Aspergillus oryzae*	XylB	BAE55732	GH43	7	30	[8]
*Penicillium herquei*	S2	AB093564	GH43	6.5	30	[9]
*Fusarium graminearum*	XyloA	ESU18126	GH43	6	30	[5]
*Bacillus pumilus*	XYL	AAC97375	GH43	7	30	[10]
*Rumen metagenome*	RuBG3A	ADM89626	GH3	7.5	35	[11]

**Table 2:** Comparison of amino acid composition and hydrogen bonds of psychrophilic xylosidase AX543 and its thermophilic counterparts Xyl43A and PtXyl43.

Enzyme	AX543	Xyl43A	PtXyl43
Microbial source	*Acremonium* sp. WCQ6A	*Humicola insolens*	*Paecilomyces thermophila*
Optimal pH	6	6.5	7
Optimal temperature (°C)	25	50	55
Relative activity at 10 °C	20%	ND	ND
Relative activity at 0 °C	5%	ND	ND
Accession No.	ON730957	KC962400	GU937001
Amino acid identity (%) with AX543	100	80	67
Glycine (%)	8.95	8.87	7.99
Proline (%)	6.48	7.03	8.58
Arginine (%)	2.47	3.36	4.73
Arginine/Lysine ratio	0.36	0.55	1.07
Numbers of hydrogen bonds	221	229	215
Numbers of hydrogen bonds with arginine	8	11	12

**Table 3:** The kinetic parameters of AX543 and mutants L2, G110S, and Q201R.

Enzyme	*K*_m_ (mM)	*V*_max_ (μmol/min·mg)	*K*_cat_ (1/s)	*K*_cat_/*K*_m_ (1/s·mM)
AX543	0.85 ± 0.057	5.63 ± 0.566	3.94 ± 0.396	4.64 ± 0.481
L2	1.49 ± 0.063	17.28 ± 0.301	12.10 ± 0.211	8.12 ± 0.256
Q201R	1.46 ± 0.005	20.20 ± 0.057	14.14 ± 0.04	9.49 ± 0.048
G110S	1.24 ± 0.042	12.80 ± 0.280	8.96 ± 0.196	7.23 ± 0.238

**Table 4:** The kinetic parameters of AX543 and its chimeras.

Enzyme	*K*_m_ (mM)	*V*_max_ (μmol/min·mg)	*K*_cat_ (1/s)	*K*_cat_/*K*_m_ (1/s·mM)
AX543	0.85 ± 0.057	5.63 ± 0.566	3.94 ± 0.396	4.64 ± 0.481
R1	1.25 ± 0.048	3.57 ± 0.069	2.50 ± 0.049	2.00 ± 0.059
R2	1.17 ± 0.033	14.07 ± 0.196	9.85 ± 0.137	8.42 ± 0.166
R3	1.00 ± 0.025	10.44 ± 0.121	7.31 ± 0.085	7.31 ± 0.103
R4	1.84 ± 0.106	10.93 ± 0.366	7.65 ± 0.256	4.16 ± 0.311
R5	1.21 ± 0.056	23.51 ± 0.552	16.46 ± 0.386	13.59 ± 0.469
R6	1.19 ± 0.034	23.20 ± 0.336	16.24 ± 0.235	13.64 ± 0.286
R7	1.63 ± 0.060	37.74 ± 0.772	26.42 ± 0.540	16.22 ± 0.656
R8	1.30 ± 0.192	64.63 ± 4.915	45.24 ± 3.441	34.72 ± 4.178
R9	1.32 ± 0.059	61.83 ± 1.463	43.28 ± 1.005	32.84 ± 1.221
R10	1.87 ± 0.044	103.20 ± 1.412	72.24 ± 0.988	38.64 ± 1.200

**Table 5:** RMSD values between the protein structures of variants and wide type AX543 and Xyl43A.

Enzyme	R1	R2	R3	R4	R5	R6	R7	R8	R9	R10
AX543	0.023	0.004	0.071	0.022	0.025	0.130	0.080	0.013	0.085	0.111
Xyl43A	0.063	0.070	0.015	0.063	0.057	0.085	0.019	0.062	0.022	0.055

## Data Availability

The data presented in this study are available within the article.

## Supplementary Materials

The following supporting information can be downloaded at: https://www.mdpi.com/article/10.3390/foods11162463/s1, Table S1: The primers used in this study.
