# Supplementary material for: Improving the Specific Activity and Thermostability of Psychrophilic Xylosidase AX543 by Comparative Mutagenesis
Source: Foods. 2022 Aug 16;11(16):2463. doi: 10.3390/foods11162463 (PMC9407119; doi:10.3390/foods11162463)
Supplement: Supplementary file 1 [file foods-11-02463-s001.zip › foods-1802214-supplementary.pdf]

Table S1 The primers used in this study.

Primer	Primer Sequence（5'-3'）
AX543-F	GGGGAATTCATGCCGCCCCTCATTACCTCC
AX543-R	CCGCTCGAGAATCTGCCTTTGTCGTGATGATCCTACC
Xyl43A-F	GACGAATTCATGGCTCCATTGATCACCAAC
Xyl43A-R	CCGCTCGAGATTCAGGTTTTTCAGTAACAATTTTTCC
Q201R-F	GCCAGCCCGAGAGCTCCTCATTC
Q201R-R	ATGAGGAGCTCTCGGGCTGGCGAC
K285R-F	CGAGTACAAGGGCAGGACATATCTCTTCTTC
K285R-R	GAAGAAGAGATATGTCCTGCCCTTGTACTCG
G110S-F	GTGGCCGTGAGTGACAAGCCCG
G110S-R	CGGGCTTGTCACTCACGGCCAC
G216A-F	GAACCCATTCTCGCCGACGACCAC
G216A-R	GTGGTCGTCGGCGAGAATGGGTTC
A119P-F	CTTCACCCCAGACCCAGAGCCCATC
A119P-R	GCTCTGGGTCTGGGGTGAAGGGG
K125P-F	CAGAGCCCATCCCAGGCAGCTACTCC
K125P-R	GGAGTAGCTGCCTGGGATGGGCTCTG
L1-F	CGCGAGACGGACATTCAATTCAACGACAATG
L1-R	CATTGTCGTTGAATTGAATGTCCGTCTCGCG
L2-F	CTCGACTCCCTCGACCCCCCTTCCGAGGTGGACCGACCACGGCGTC
L2-R	GGGGGGTCGAGGGAGTCGAGGGAGAAGACATGGTAGTCGGCCATGTC

L3-F	CCCAAGGAACCCAGCGGCTCCGGGGCCAAGGCGCTCGGCCCTCGCGCCGCCAAGTTGACAG
L3-R	GAGCCGCTGGGTTCCTTGGGGCCGGACCATTCCGCGTCGAACGTGTCGTCGCCCTTTTGGTAGC
L4-F	TACCATGTCTTCTCCCTCGACTCCCTGACC
L4-R	GTGGTCGGTCACCTCGGAAGGGGGGTCGA
